# A 26-GHz transmitter front-end using double quadrature architecture

**DOI:** 10.1371/journal.pone.0216474

**Published:** 2019-05-23

**Authors:** Hyo-Sung Lee, Mingyo Park, Byung-Wook Min

**Affiliations:** 1 School of Electrical and Electronic Engineering, Yonsei University, Seoul 03722, Republic of Korea; 2 School of Electrical and Computer Engineering, Georgia Institute of Technology, Atlanta, GA, United States of America; Instituto Nacional de Astrofisica Optica y Electronica, MEXICO

## Abstract

A 26-GHz transmitter front-end is designed using 65 nm CMOS technology. The double frequency conversion transmitter consists of an intermediate frequency(IF) mixer, an millimeter-wave(mm-wave) mixer, and a pre-power amplifier. A double quadrature architecture is employed to accomplish image rejection without using an image rejection filter for the first time in the mm-wave frequency band. The IF mixer cores are designed as harmonic rejection mixers to avoid using IF filters. The measured conversion gain is 26.85±0.65 dB, with LO_2_ (IF LO) at 1–1.5 GHz and 26.9±0.6 dB with LO_1_ (mm-wave LO) at 27–29 GHz. The measured output return loss is less than -10 dB at 25.7–27.2 GHz. The output 1-dB compression point and the saturation output power measured at 26 GHz are 10 dBm and 14.1 dBm, respectively. The output-referred third-order intercept point (OIP3) measured at 26 GHz is 15.76 dBm. The third-order distortion, suppressed by the harmonic rejection mixer, is -60.5 dBc at an output power of 10 dBm. The error vector magnitude measured for OFDM 16-QAM with a 110-MHz signal bandwidth is -17.7 dB at an average output power of 3.5 dBm. The total power consumption of the proposed 26-GHz transmitter front-end is 267 mW, and it occupies a chip area of 2.31 mm^2^.

## Introduction

With the explosive increase of wireless data capacity, the demand for fifth generation (5G) wireless communication systems has been increasing in recent years [1, 2]. One of the key goals of 5G communications is to procure a wide bandwidth, because they require high data rates. However, it is difficult to secure a wide bandwidth at the existing fourth generation (4G) communication frequencies (below 6 GHz), and future standard 5G communication frequencies are expected to be millimeter wave (mm-wave), in order to procure a wide bandwidth [3–5]. In particular, the 26 GHz spectrum is a promising candidate for 5G wireless communications in Europe and China.

**Fig 1:**
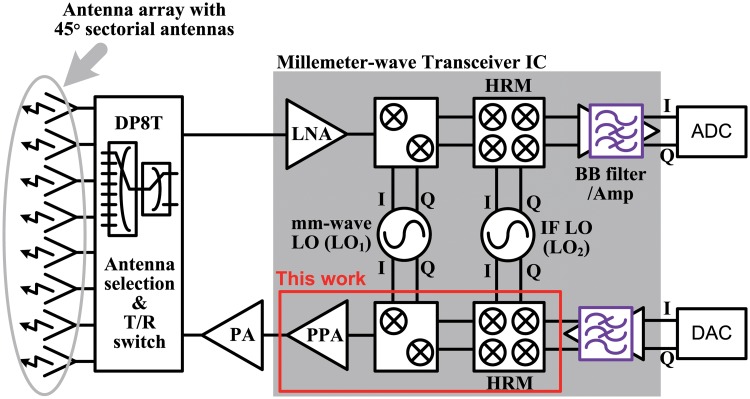
Block diagram of a switched beam selection system for 5G communication system for UE.

Millimeter waves, including those in the 26-GHz spectrum, inherently suffer from significant propagation loss and less diffraction compared with 4G communication frequencies, which are below 6 GHz. Fig 1 presents a block diagram of a mm-wave switched beam system for 5G communications in UE. Eight high-gain antennas with fixed beams are placed on a circle and can cover a 360° azimuth plane [6]. This system does not require phase shifters because beam selection is performed without beam scanning. The UE can select the optimum beam using antenna selection switches. Antenna selection switches and a T/R switch can be integrated as a double-pole eight-throw (DP8T) switch. A double frequency conversion structure was adopted because it has lower local oscillator (LO) feedthrough and LO pulling than the direct-conversion structure. This allows for channel tuning at intermediate frequency (IF). A power amplifier (PA) with power combining techniques was excluded from this design because it would require very high heat dissipation and take up a lot of die area [7, 8]. Instead, a pre-power amplifier (PPA) with a medium output power was implemented to meet the linearity requirement of the system.

In this work, a 26-GHz superheterodyne transmitter (TX) front-end for UE was designed using a 65-nm CMOS process. A double quadrature architecture was adopted to reject the image signal without using an image rejection (IR) filter. This enables the integration of a PPA. The IF mixer cores were designed as harmonic rejection mixers (HRMs) to avoid using IF filters for the first time in mm-wave transmitters, and achieve a high integration in the proposed transmitter. In Section II, typical transmitter architectures are discussed and the need for the double quadrature architecture with HRMs is explained. Section III describes the circuit design for the 26-GHz transmitter front-end in detail, followed by our simulated and measured results, presented in Section IV.

## Transmitter architecture

A transmitter based on an I/Q modulator can be classified either as a direct-conversion (homodyne) transmitter or an indirect-conversion (superheterodyne) transmitter. Direct-conversion transmitters perform frequency up-conversion only once, and therefore have a simpler structure than indirect-conversion transmitters and take up a smaller die area. However, direct-conversion transmitters have some disadvantages which stand out in mm-wave communication systems for 5G. First, LO pulling due to the high output power of the PA occurs because the transmit signal frequency and the LO frequency are the same. This distorts the oscillation spectrum and degrades the phase noise performance of the system [9]. Secondly, because the desired I/Q modulator has to be designed for mm-waves, carrier leakage due to parasitic elements is relatively large. In contrast, indirect-conversion transmitters, which generally perform a two-step (or more) frequency translation, can be used to alleviate the problems of the direct-conversion transmitter. However, multiple off-chip filters are usually required because many harmonics and image signals are generated during frequency mixing. These filters increase the complexity of the transmitter architecture. In this chapter, the typical indirect-conversion transmitter architecture is discussed, and a modified transmitter with a double quadrature architecture and HRMs is described.

### Indirect-conversion transmitter

**Fig 2:**
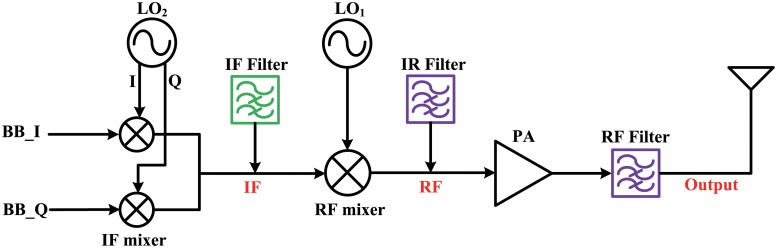
Block diagram of general double-conversion transmitter.

**Fig 3:**
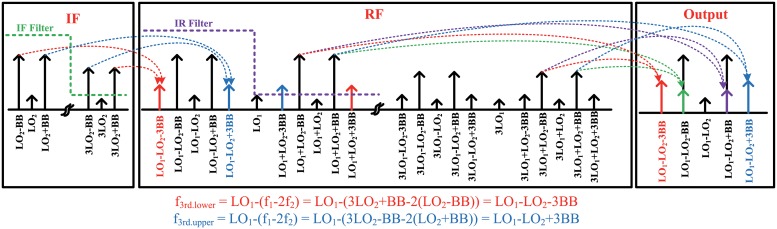
Frequency domain analysis for a double-conversion transmitter.

A general double frequency conversion transmitter is shown in Fig 2. The baseband signals are up-converted by an I/Q modulator (IF mixer) to the IF. The IF signals form a constellation and that are up-converted to RF by the RF mixer. The I/Q modulator and the RF mixer, which are typically designed as a switching mixer, are very nonlinear devices. Therefore, the IF signals intermodulate with the third-order harmonics of the I/Q modulator at the RF mixer (Fig 3). These intermodulations (f_3*rd*.*lower*_, f_3*rd*.*upper*_) are located in the in-band and cannot be removed by subsequent filters. Therefore, the IF filter after the I/Q modulator is essential for suppressing third-order intermodulation in typical double frequency conversion transmitters. An image rejection (IR) filter located next to the RF mixer is also required. Image signals not only act as spurious signals, but also intermodulate with the third-order harmonics of the RF mixer in a non-linear PA (Fig 3). Because those intermodulations are also located in the in-band, they cannot be removed by subsequent filters. Hence, an IF filter and an IR filter are necessary in indirect-conversion transmitters. However, these filters are usually designed using discrete components or occupy a huge die area on the chip. The following chapters explain how to eliminate these intermodulations without using filters.

### Double quadrature architecture

**Fig 4:**
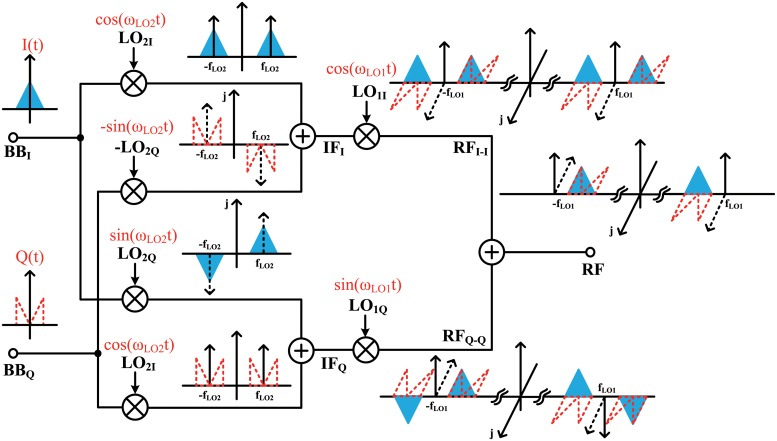
Frequency domain interpretation of a double quadrature architecture for the baseband I and Q channels.

Image rejection methods without using filters have been developed by Hartley and Weaver [10, 11]. The Weaver architecture is suitable for indirect-conversion transmitters because it requires a two-step frequency conversion. The double Weaver architectures for I/Q modulation can be modified to a double quadrature architecture, as shown in Fig 4 [12]. The double quadrature architecture shares the signal path of the IF in order to reduce two RF mixer cores, in contrast with the double Weaver architecture. The baseband I (BB_*I*_) and Q (BB_*Q*_) signals are multiplied by the in-phase and quadrature LO_2_ signals and then up-converted to the IF. The four up-converted signals are summed into the in-phase and quadrature IF signals (IF_*I*_ and IF_*Q*_), which can be expressed as

$$\begin{array}{c} \begin{array}{c} IF_{I}=BB_{I}\cdot LO_{2I}+BB_{Q}\cdot \left(-LO_{2Q}\right)\\ =I\left(t\right)\cos\left(\omega_{LO2}\right)t-Q\left(t\right)\sin\left(\omega_{LO2}\right)t \end{array} \end{array}$$ (1)

$$\begin{array}{c} \begin{array}{c} IF_{Q}=BB_{I}\cdot LO_{2Q}+BB_{Q}\cdot LO_{2I}\\ =I\left(t\right)\sin\left(\omega_{LO2}\right)t+Q\left(t\right)\cos\left(\omega_{LO2}\right)t. \end{array} \end{array}$$ (2)

Each IF_*I*_ and IF_*Q*_ signal is multiplied by the in-phase and quadrature LO_1_ signals, respectively, and they are then up-converted to RF_*I*−*I*_ and RF_*Q*−*Q*_. When RF_*I*−*I*_ and RF_*Q*−*Q*_ are combined, image rejection and I/Q modulation occurs. The final RF signal can be expressed as

$$\begin{array}{c} \begin{array}{cc} & RF=IF_{I}\cdot LO_{1I}+IF_{Q}\cdot LO_{1Q}\\ & =I\left(t\right)\cos\left(\omega_{LO1}-\omega_{LO2}\right)t+Q\left(t\right)\sin\left(\omega_{LO1}-\omega_{LO2}\right)t. \end{array} \end{array}$$ (3)

The image signals, which correspond to the (*ω*_*LO*1_ + *ω*_*LO*2_) terms, are finally rejected while maintaining a wider bandwidth than when using an IR filter [13]. I(t) and Q(t) are modulated by cos(*ω*_*LO*1_—*ω*_*LO*2_)t and sin(*ω*_*LO*1_—*ω*_*LO*2_)t, respectively. In this work, the lower side band (LSB) contains the desired signals and the upper side band (USB) is rejected, as it contains the image signals.

### Harmonic rejection technique

The double quadrature architecture removes the requirement for an IR filter, but an IF filter is still needed to reduce the harmonics of the IF mixer. The third-order harmonics generated by the nonlinear IF mixer intermodulate with the fundamental tones in the nonlinear RF mixer (Fig 3). These intermodulations degrade the modulation accuracy and cannot be eliminated with filters. Therefore, the third-order harmonics of the IF mixer should be removed before the RF mixer. In this work, an IF mixer with a harmonic rejection technique was designed to reject the third-order harmonics of the IF mixer without using IF filters.

**Fig 5:**
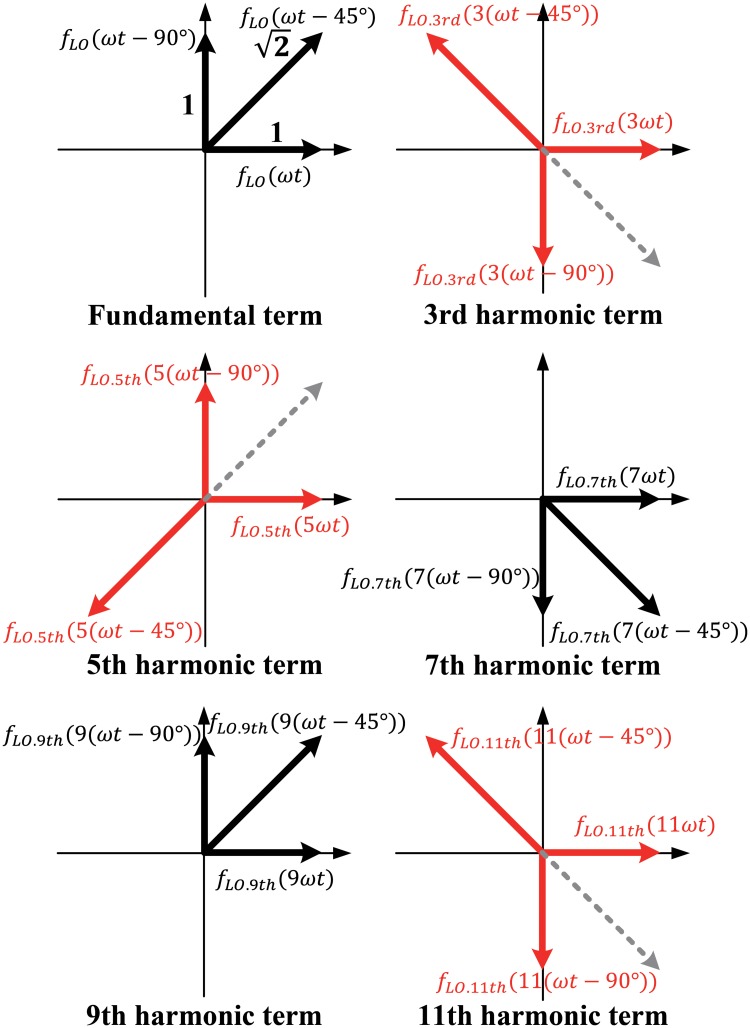
Vector sum diagram of the harmonic rejection technique.

The proposed harmonic rejection technique can be simply described by the vector sum diagram shown in Fig 5. The phases of the three LO signals are 45° apart from each other (0°, 45°, 90°). The third-order harmonic is ideally canceled when the three LO signals are summed at an amplitude rate of $1:\sqrt{2}:1$. The phases of the third-order harmonics of the LO signals are three times the phases of the fundamental LO signals, i.e., 0°(0° × 3), 135°(45° × 3) and 270°(90° × 3)[14]. The fifth-order harmonic and the eleventh-order harmonic are also canceled in the same way. However, the seventh-order and ninth-order harmonics are $2\sqrt{2}$ times bigger than when using only one LO signal. The amplitudes of these harmonics are relatively low, so they can be disregarded.

**Fig 6:**
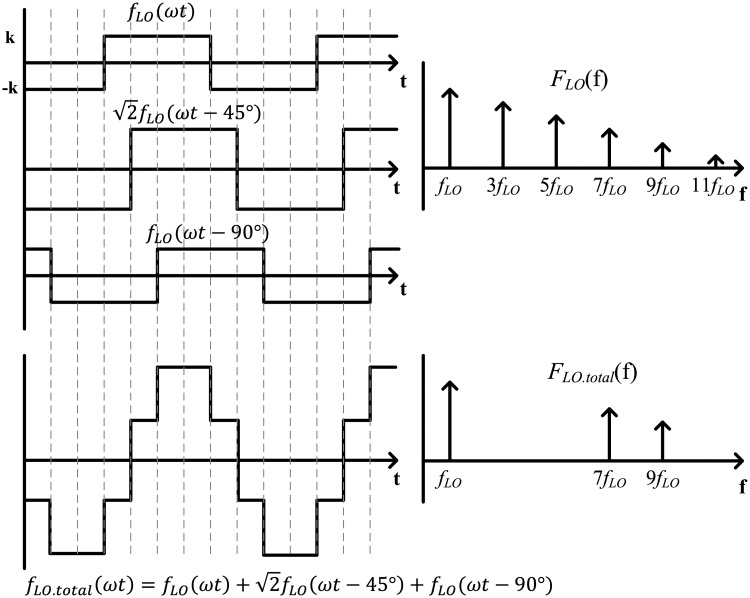
Three-phase LO square waves and the final quantized sinusoid LO signal.

The three LO square waves (0°, 45°, 90°) and the final quantized sinusoid LO signal are shown in Fig 6. The final quantized sinusoid LO signal, *f*_*LO*.*total*_(*ω*t), can be generated by adding the three-phase square waves. The three square waves are summed at an amplitude rate of $1:\sqrt{2}:1$ to achieve the aforementioned perfect cancellation. The third and fifth harmonic elimination can be confirmed mathematically using the Fourier series representation of the signals. The Fourier series representation of a square wave, *f*_*LO*_(*ω*t), is given by

$$\begin{array}{c} f_{LO}\left(\omega t\right)=A\sum_{n=1}^{\infty }\frac{1}{2n-1}\sin\left(\left(2n-1\right)\omega t\right) \end{array}$$ (4)

where *A* is 4*k*/*π* and *k* is the absolute value of the amplitude of the square wave. Square waves do not have even harmonics. The general expression for the sum of three square waves with phase intervals of 45° and with amplitudes of 1, $\sqrt{2}$, and 1 is given by

$$\begin{array}{c} \begin{array}{cc} f & {}_{LO.total}\left(\omega t\right)\\ & \;\;\;\;\;\;\;\;\;\;\;\;\;\;\;=f_{LO}\left(\omega t\right)+\sqrt{2}f_{LO}\left(\omega t-\frac{\pi }{4}\right)+f_{LO}\left(\omega t-\frac{\pi }{2}\right)\\ & \;\;\;\;\;\;\;\;\;\;\;\;\;\;\;=2\sqrt{2}A\sum_{n=1}^{\infty }[\frac{1}{8n-7}\sin\left(\left(8n-7\right)\omega t-\frac{\pi }{4}\right)\\ & \;\;\;\;\;\;\;\;\;\;\;\;\;\;\;\;\;\;\;\;\;\;\;\;\;\;\;\;\;\;\;\;\;\;\;\;\;\;\;\;\;+\frac{1}{8n-1}\sin\left(\left(8n-1\right)\omega t+\frac{\pi }{4}\right)] \end{array} \end{array}$$ (5)

The square wave that is shifted by 45° is scaled by $\sqrt{2}$ to achieve a perfect cancellation of the (8n-5)th and (8n-3)th harmonics. The (8n-7)th tones, including the fundamental tone, and the (8n-1)th tones are $2\sqrt{2}$ times bigger than when using a single square wave. The next challenge lies in how to combine the three square waves. They can be combined using current-commutating switching mixers. The Gilbert cell active mixer, which is a typical current-commutating mixer, can combine the three IF current signals. Further details are given in section III-A.

## Details of transmitter front-end

**Fig 7:**
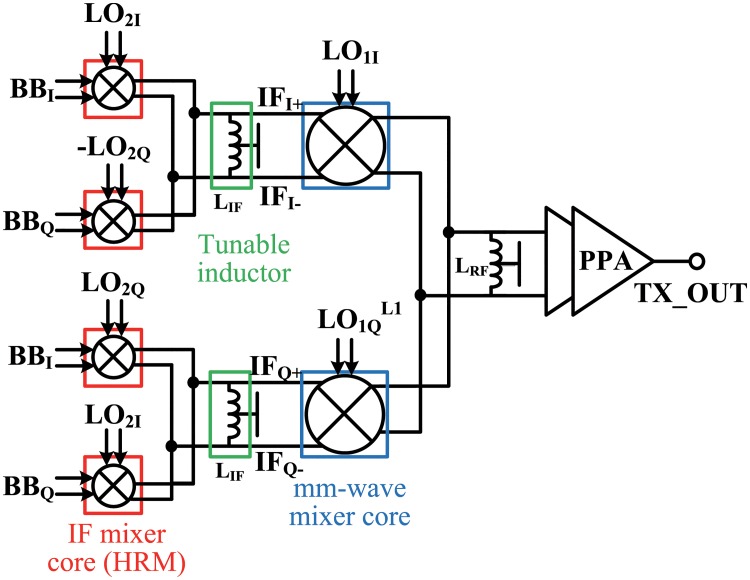
Block diagram of a 26-GHz transmitter front-end.

The block diagram of the proposed 26-GHz transmitter front-end is shown in Fig 7. The double quadrature architecture was adopted to achieve wide-band image rejection without using an IR filter. By not employing an IR filter, which are generally designed off-chip, PPA integration was possible. The 26-GHz transmitter front-end consists of four IF mixer cores, two mm-wave mixer cores, and a two-stage cascode PPA. The IF mixer cores were designed as HRMs avoid the need for IF filters. The IF mixer cores and the mm-wave mixer cores were fully differential Gilbert cell active mixers. Because flicker noise is not an important issue in the transmitter, the active mixer was suitable for performing the frequency conversion of the transmitter. Conventional differential active mixer cores for high frequencies commonly use two load inductors. The proposed architecture had six differential active mixer cores, and therefore twelve load inductors were required. In this architecture, all load inductors were designed as symmetrical differential spiral inductors with a center tap to reduce the die area occupied by them. In addition, each pair of mixer cores shared a load inductor. Thus, the number of load inductors required was reduced to three. Because the IF spectrum should be wide enough for wide band mm-wave communication, the load inductors (L_*IF*_) of the IF mixer were designed as low-Q tunable inductors for a selected channel. All circuits were designed to be fully differential except for the output stage of the PPA. A transformer balun was used at the PPA output to convert the differential signal into an unbalanced signal.

### IF mixer (harmonic rejection mixer)

**Fig 8:**
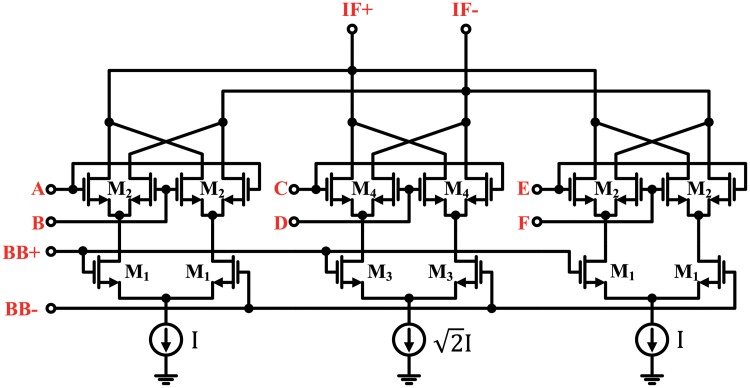
Schematic of harmonic rejection mixer core.

The IF mixer cores were designed as an HRM using the harmonic rejection technique introduced in Section II-C. As mentioned before, an important point in this harmonic rejection technique is how to combine the three square waves. Three square waves with 45° phase intervals can be summed via current summation in the current-commutating mixers. The HRM consists of three Gilbert cell active sub-mixers, as shown in Fig 8, which are typical current commutating mixers [15]. The baseband voltage signals were amplified in the transconductance stages (M1, M3) and converted into current signals. These baseband current signals were mixed with three square waves at the switching stages (M2, M4) and up-converted to IF current signals. These up-converted current signals still maintained a 45° phase interval. The three IF current signals were added and the (8n-5)th and (8n-3)th harmonics were canceled. To achieve a perfect harmonic cancellation, the sizes of the transconductance stage (M3) and the current source in the middle sub-mixer were scaled by $\sqrt{2}$ [16]. The gate widths of M1, M2, M3, and M4 were 788, 640, 1075.2, 896 *μ*m, respectively, and the gate lengths of all transistors were 60 nm.

**Fig 9:**
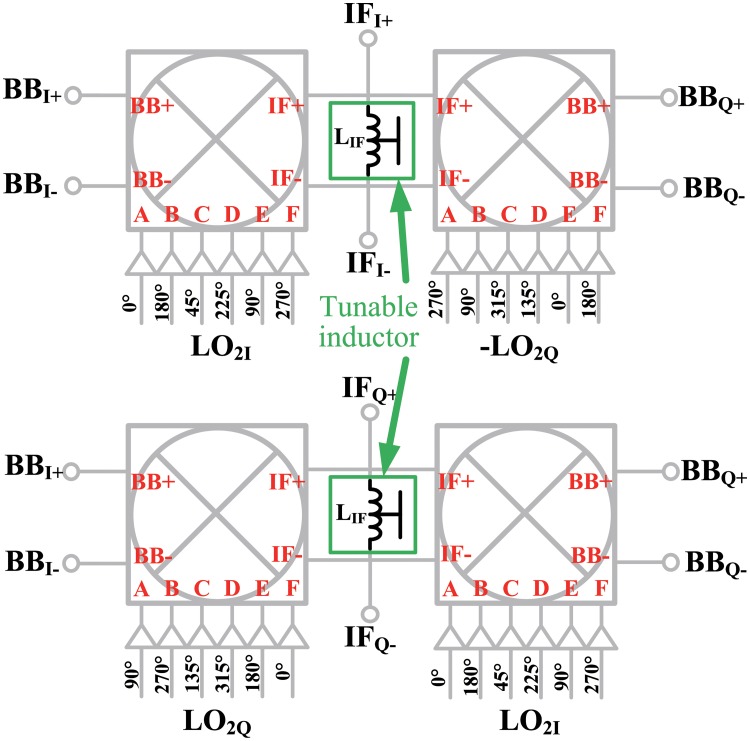
IF mixer with eight phases of LO signals.

As shown in Fig 7, the double quadrature architecture requires four IF mixer cores and I/Q LO signals. The phases of the differential in-phase LO signals required by the HRM were <0°, 180°>, <45°, 225°>, and <90°, 270°>, and the phases of the differential quadrature LO signals were <90°, 270°>, <135°, 315°>, and <180°, 0°>. Therefore, the IF mixer required eight LO signals with a 45° phase interval. The block diagram of the IF mixer with eight LO signals is shown in Fig 9. In order to reject the USB, an arrangement of the eight LO signals and the way of summation of the IF signals were determined. The LO buffers were designed as general inverter-type amplifiers, and six buffers were used for each IF mixer core. Each pair of IF mixer cores shared a load inductor to reduce the chip area occupied by them.

**Fig 10:**
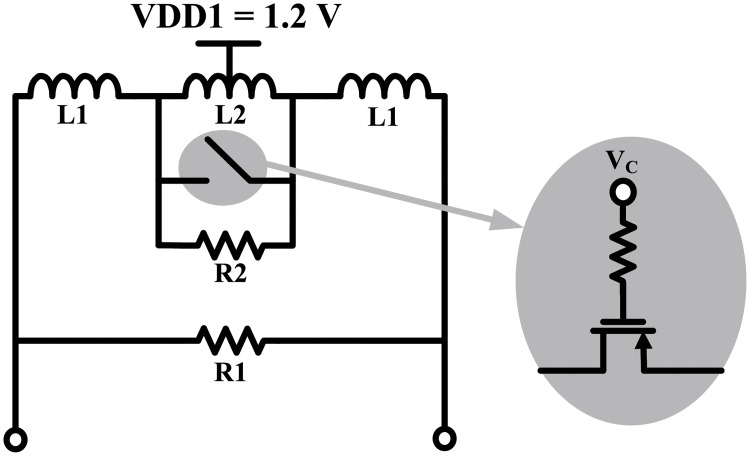
Tunable inductor.

Extremely high data rates can be achieved in mm-wave communication systems by using a wide bandwidth. Therefore, in indirect-conversion transmitters, the IF should also have a wide bandwidth. In order to ensure a wide bandwidth at the IF, the load inductors were designed as low-Q tunable inductors. Fig 10 shows a schematic of the proposed low-Q tunable inductors. A switch was used to control the inductance by symmetrically connecting the two tabs of the inductor. When the switch is open, the inductance is 2L1+L2 and the IF mixer operates in low-frequency mode. When the switch is closed, the inductance is 2L1 and the IF mixer operates in high-frequency mode. The switch was designed as a PMOS transistor because the voltage at the source and drain of the switch is VDD1 (1.2 V). Resistor R1 was connected in parallel with the entire inductor to reduce the Q factor of the inductor, and resistor R2 was connected in parallel with the switch to reduce the Q factor difference between the two modes. The inductors were optimized with a 2.5-D full wave electromagnetic simulator (Sonnet), and the extracted S-parameters were used in a circuit simulator.

### Millimeter-wave mixer

**Fig 11:**
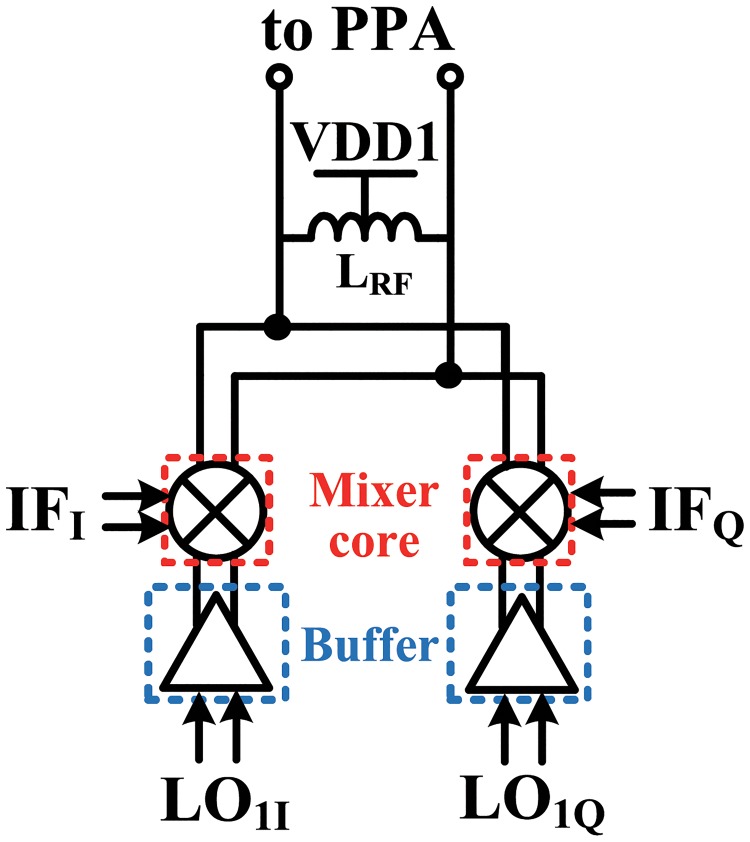
Mm-wave mixer block diagram for image rejection.

**Fig 12:**
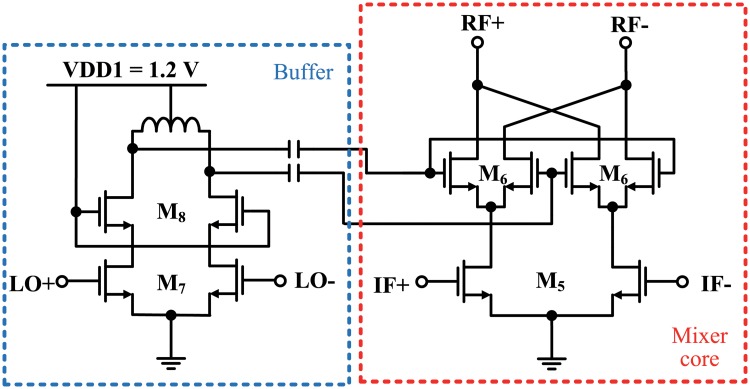
Schematic of the mm-wave mixer core and buffer.

Fig 11 shows a block diagram of the millimeter-wave mixer that converts the IF to mm-wave frequency. The mm-wave mixer consisted of two double-balanced Gilbert cell active mixer cores, two differential LO buffers, and a load inductor. Because the gain of the IF mixer with low-Q inductors is low (0±1.5 dB) to cover a wide IF band, the mm-wave mixer should have a high gain. The linearity of the mm-wave mixer should also be considered, because it must deliver large power to the input of the PPA. For these reasons, the mm-wave mixer cores were implemented as active mixers to satisfy the requirements of high gain and power handling capacity. A schematic of the mm-wave mixer core and buffer is shown in Fig 12. The current source under M5 was not used in order to maximize the conversion gain of the mm-wave mixer and to alleviate voltage headroom. In order to reduce a chip area, the mm-wave mixer cores shared a load inductor, like the IF mixer cores. The LO buffers for the mm-wave mixer consisted of differential cascode amplifiers (M7, M8) with load inductors, because the gain of the inverter type buffers is generally low in mm-wave frequencies. Due to the double quadrature architecture, image rejection occurred at the output of the mm-wave mixer, and the need for an IR filter was eliminated. Therefore, integration density was increased because the output of the mm-wave mixer could be connected directly to the input of the PPA. The gate widths of M5, M6, M7, and M8 were 312, 64, 88, 88 *μ*m, respectively, and the gate lengths of all transistors were 60 nm. The inductors for the mm-wave mixer and buffers were also optimized using an electromagnetic simulator (Sonnet) and the extracted S-parameters were used in a circuit simulator.

### Pre-power amplifier

**Fig 13:**
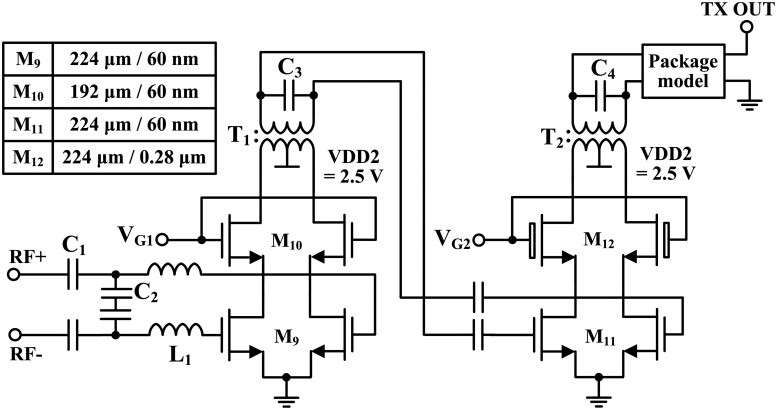
Schematic of pre-power amplifier.

The double quadrature architecture used allowed for a PPA to be implemented right next to the mm-wave mixer. Fig 13 shows the schematic of the PPA. In order to achieve a higher output power and a better frequency response than those of the common-source configuration, the PPA consisted of a two-stage cascode amplifier, which provided good reverse isolation and a high voltage swing [17]. The first stage was designed as a driver stage to provide high gain, while the second stage was designed as a power stage to achieve the required high output power. In the common gate stage (M_12_) of the second stage, 2.5-V thick-oxide high-voltage transistors with a gate length of 0.28 *μ*m were used to endure the high voltage swings. The other transistors (M_9_, M_10_ and M_11_) had a gate length of 65 nm in order to maximize the PPA gain. V_*G*1_ and V_*G*2_ were 2.1 V and 1.9 V, respectively. The V_*DS*_ of all 65-nm transistors was simulated and checked so as to not exceed the maximum allowed voltage (1.32 V) of 65-nm transistors [7]. A load-pull simulation was performed in the second stage to determine the optimal load impedance for high output power and sufficient gain while varying the sizes of the transistors and current density [17, 18]. The input matching network of the PPA performed conjugate matching with the output impedance of the mm-wave mixer, (100 + j53). Two series inductors (L_1_) and two parallel capacitors (C_2_) were employed in the matching network. The series LC network is composed of a series inductor (L_1_), and the gate-source capacitance (C_*GS*_) of M_9_ boosted the input voltage with its quality factor [19]. C_3_ and C_4_ were implemented at the inter-stages and in the output matching network.

**Fig 14:**
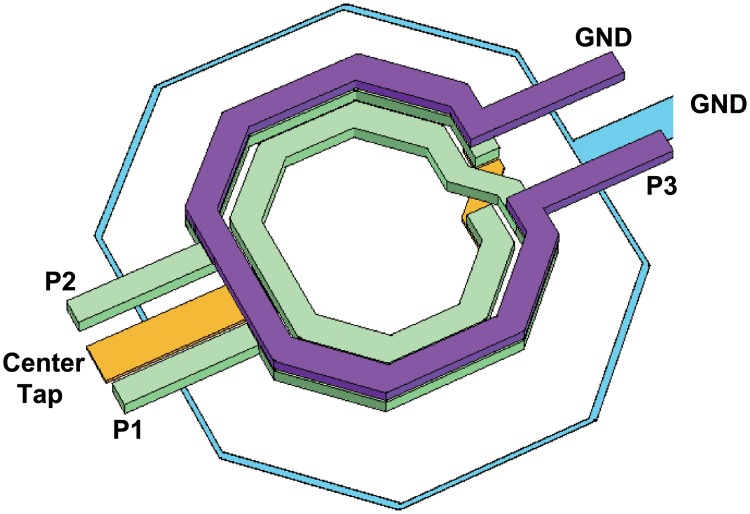
Physical layout of the transformer balun.

**Fig 15:**
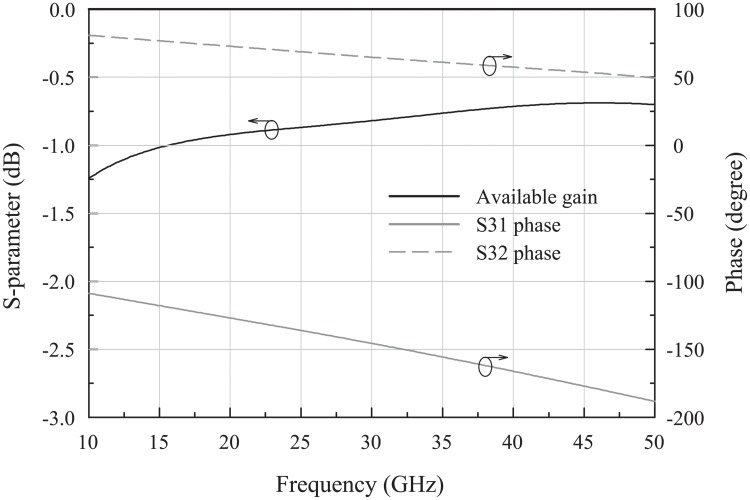
Simulated insertion loss of the transformer balun.

Transformers were employed as the loads of the PPA and had a wider frequency response and a simpler routing than the inductors. The transformers were implemented in the two top metal layers to maximize the coupling and quality factors. As shown in Fig 14, the transformer in the second stage (T_2_) acts as a balun that converts the differential signal into a single-ended one. The transformer balun had a good phase balance and was designed with wide and thick copper (width: 5 *μ*m, thickness: 3 *μ*m) to endure large current flow due to the high output power of the PPA. The simulated available gain of the transformer balun was -0.8 dB at 26 GHz, and the phase difference was well maintained at 180° in mm-wave frequencies (Fig 15). All transformers (T_1_, T_2_) and inductors (L_1_) were designed with an electromagnetic simulation tool (Sonnet).

## Measurement results

**Fig 16:**
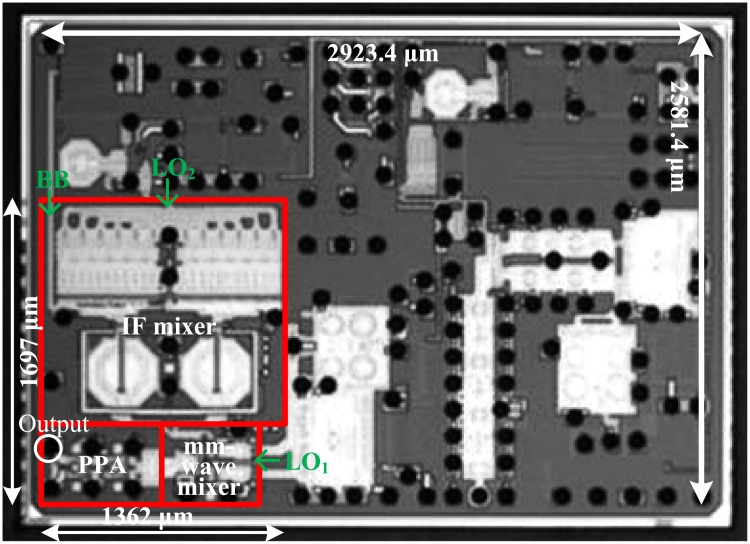
Microphotograph of the mm-wave transceiver.

**Fig 17:**
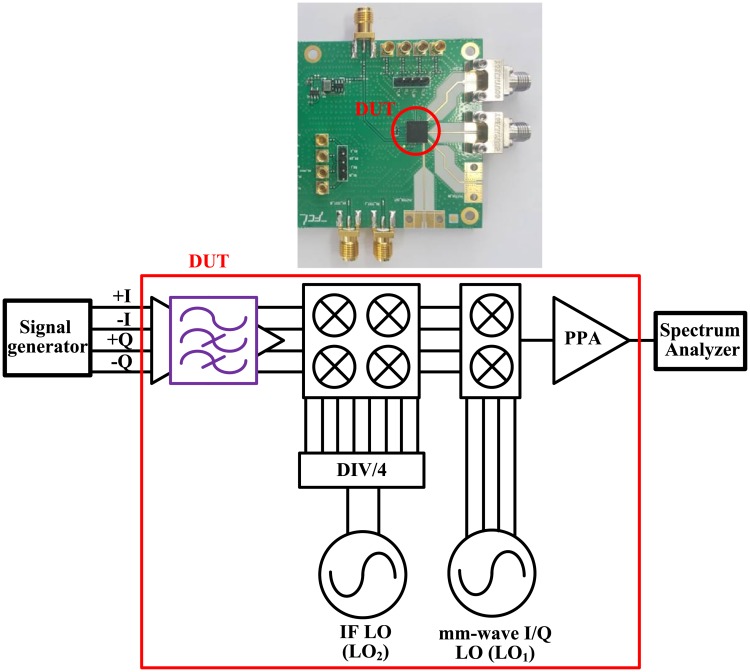
Measurement setup for the 26-GHz transmitter front-end.

The 26-GHz transmitter front-end was implemented using a 65-nm CMOS process. A microphotograph of the 26-GHz transmitter front-end is shown in Fig 16; the size of the transmitter was 1397 × 1697 *μ*m^2^. A receiver front-end, a baseband filter, a programmable amplifier (PGA), an IF phase locked loop (PLL), a frequency divider for IF LO, a mm-wave PLL, and a mm-wave I/Q voltage controlled oscillator were all implemented. The designed chip was packed as a flip-chip package that employs bump pads with a diameter of 84.4 *μ*m and a minimum spacing of 150 *μ*m. The ball-grid-array (BGA) package and the PCB layout was simulated using High Frequency Structure Simulator (HFSS). Fig 17 shows the measurement setup for the 26-GHz transmitter front-end. The loss of connectors, cables, and transmission lines on the PCB board were de-embedded and the gain of the baseband filter and PGA was set to 0 dB.

### Continuous wave measurements

The 26-GHz transmitter front-end was measured using a Keysight E4438C ESG vector signal generator and an N9030A PXA signal analyzer. The transmitter measurements were performed with a single-sideband test. The actual baseband I/Q signals were randomly different for I/Q modulation, but the phase of baseband Q signal was delayed 90° compared with the I signal in the simulations and measurements. As a result, single-sideband rejection occurred. The I/Q balance of the IF mixer was checked by measuring the single-sideband rejection ratio (SRR).

**Fig 18:**
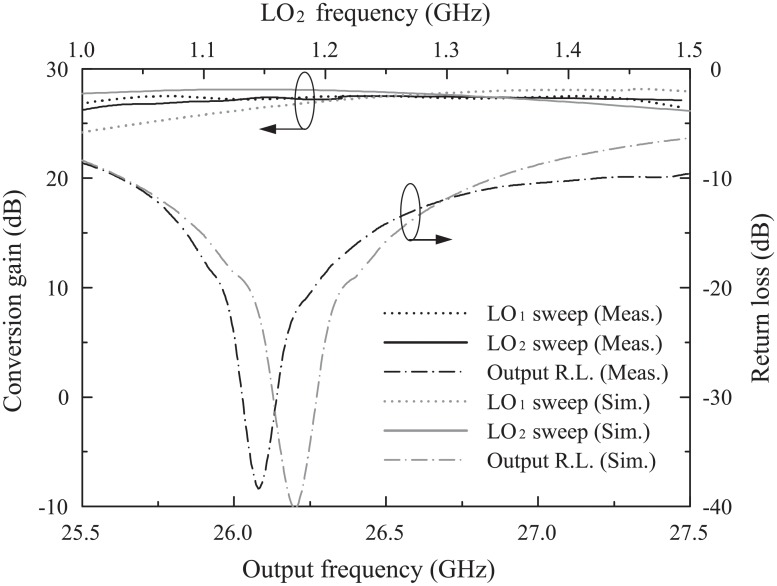
Simulated and measured voltage conversion gains and output return loss of the proposed mm-wave transmitter front-end.

Fig 18 shows the simulated and measured conversion gains and output return loss (S-parameter) of the 26-GHz transmitter front-end. Conversion gain is defined as the gain from the baseband I channel voltage to the PPA output voltage. The conversion gains were measured and simulated by varying LO_1_ and LO_2_. The measured and simulated conversion gains varying LO_2_ at 1–1.5 GHz were 26.85±0.65 dB and 27.1±1.0 dB when LO_1_ was set at 27.5 GHz and BB at 10 MHz, respectively. The measured and simulated conversion gains varying LO_1_ of 27–29 GHz were 26.9±0.6 dB and 26.1±2.0 dB when LO_2_ was set at 1.5 GHz and BB at 10 MHz, respectively. The measured output return loss were less than -10 dB at 25.7–27.2 GHz and the simulated result was less than -10 dB at 25.7–26.8 GHz. This 26-GHz transmitter front-end had a flat conversion gain and good output matching at the operating frequency.

### Linearity and harmonic spurs

**Fig 19:**
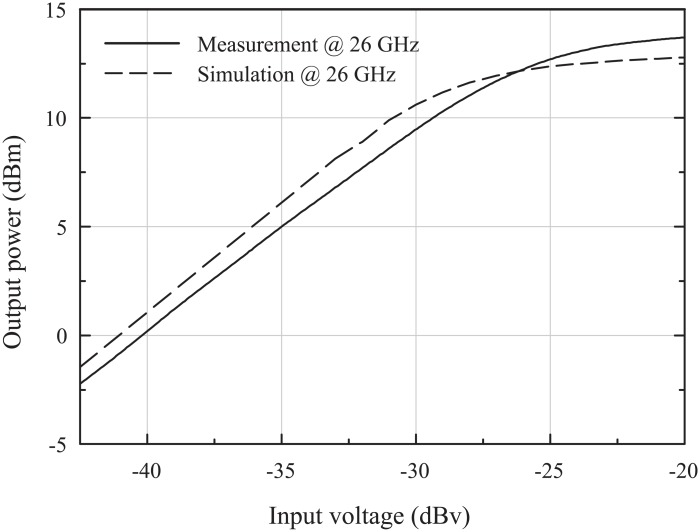
Simulation and measurement results of the output power versus input voltage.

**Fig 20:**
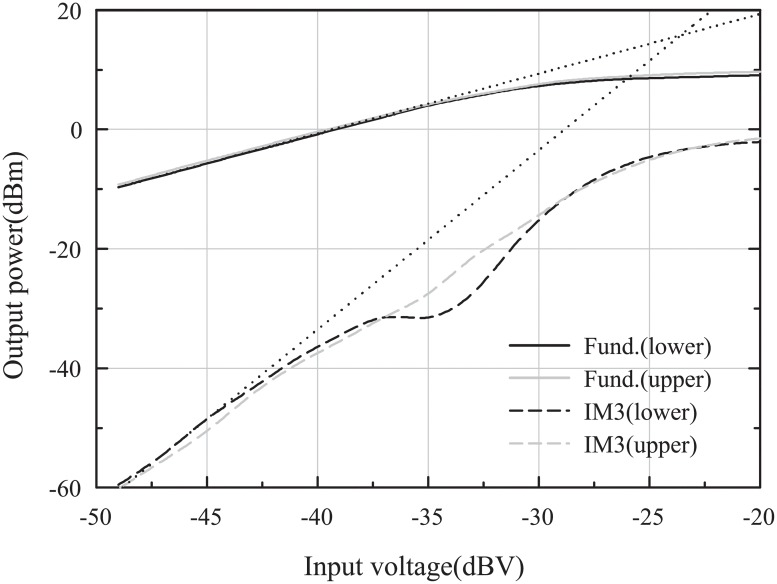
Measured third-order intermodulation distortion versus input voltage.

Fig 19 shows the measured output power versus the input voltage. The x-axis was determined as the input voltage because the input power of the IF mixer is difficult to define due to the baseband filter. The measured and simulated output-referred 1-dB compression points (OP_1*dB*_) for a BB of 10 MHz, LO_1_ at 27.5 GHz, and LO_2_ at 1.5 GHz were 10 dBm and 11.4 dBm, respectively. The measured and simulated saturation output powers (P_*SAT*_) were 14.1 dBm and 12.8 dBm, respectively. The high difference between the measured P_*SAT*_ and OP_1*dB*_ means that the PPA operates in a reliable region [7]. Fig 20 shows the measurement results of the third-order intermodulations with 12 MHz and 20 MHz baseband fundamental tones when the frequency of LO_1_ and LO_2_ were 27.5 GHz and 1.5 GHz, respectively. The measured output-referred third-order intercept point (OIP3) was 15.76 dBm, and the third-order intermodulation distortion (IMD3) was <-30 dBc when the sum of the two output powers was 4 dBm.

Table 1 summarizes the measured harmonic spurs at fundamental output power of 10 dBm (OP_1*dB*_ point). The spurious emission limit in the 26 GHz band has not yet been determined, but it should be at least 30 dBc lower than the fundamental tone, considering the 3GPP standard. The third-order distortion caused by the IF mixer was suppressed to -60.5 dBc, owing to the use of the HRM. The HRM successfully eliminated the third-order harmonic without using IF filters. LO leakages (LO_1_ and LO_1_-LO_2_) were above -30 dBc, but this was solved by adjusting the DC offset of the mixers. I/Q calibration of the mm-wave mixer and the frequency synthesizer will be necessary in future works, because the image rejection ratio (IRR) and the second order harmonic caused by the mm-wave mixer were above -30 dBc.

**Table 1:** Measured harmonic spurs.

LO_1_-(LO_2_+BB)	10 dBm	Fundamental output power
LO_1_-(LO_2_-3BB)	-60.5 dBc	3rd order distortion (HRM)
LO_1_	-21.7 dBc	mm-wave LO leakage
LO_1_-LO_2_	-20.2 dBc	IF LO leakage
LO_1_-(LO_2_-BB)	-34.8 dBc	Single-sideband rejection ratio
LO_1_+(LO_2_+BB)	-28.9 dBc	Image rejection ratio
LO_1_-3(LO_2_+BB)	-34.0 dBc	3rd harmonic (mm-wave mixer)
LO_1_-(3LO_2_+BB)	-34.4 dBc	3rd harmonic (IF mixer)
LO_1_-2(LO_2_+BB)	-28.5 dBc	2nd harmonic (mm-wave mixer)
LO_1_-(2LO_2_+BB)	-43.5 dBc	2nd harmonic (IF mixer)

### Modulated signal measurements

**Fig 21:**
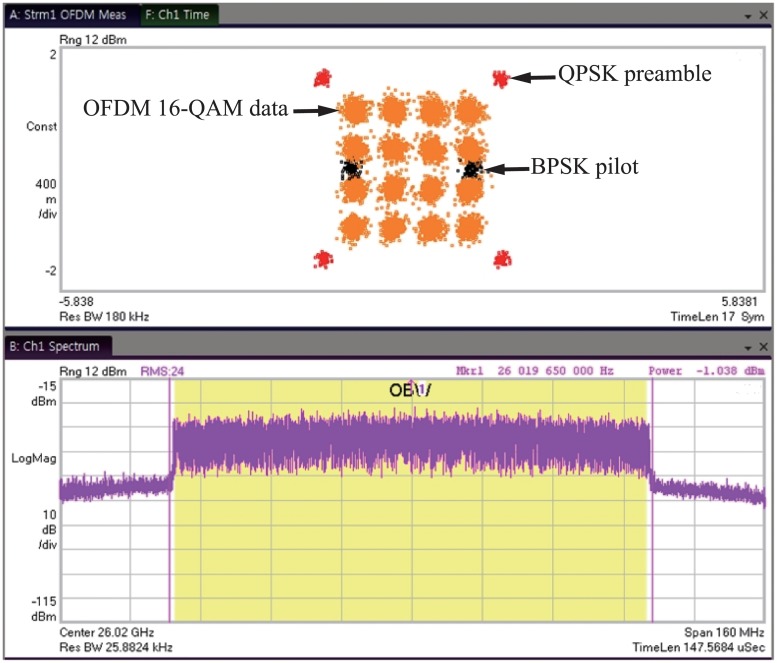
Measured constellation and spectrum for OFDM 16-QAM.

Modulated signal measurements for the 26-GHz transmitter front-end were also performed. The differential I/Q baseband signals were generated using a Keysight M8190A arbitrary waveform generator (AWG) and the up-converted and modulated signals were measured using a Keysight N9030A PXA signal analyzer. The measurement data, including the error vector magnitude (EVM), were analyzed and calculated using the Keysight 89600 vector signal analyzer (VSA) software. The measured constellation and spectra of the proposed 26-GHz transmitter front-end are shown in Fig 21. The measured EVM for orthogonal frequency division multiplexing (OFDM) 16 quadrature amplitude modulation (QAM) with a signal bandwidth of 110 MHz was -17.7 dB for an average output power (P_*AVG*_) of 3.5 dBm. The quadrature phase shift keying (QPSK) preamble and the binary phase shift keying (BPSK) pilot are also generated in the AWG to synchronize the demodulator and to compensate frequency and phase shift of the signals.

The 3.5 dBm output power of OFDM-16QAM modulation will be able to satisfy the 3GPP minimum peak EIRP specification of 5G mm-wave handheld UE at n257(28 GHz) and n258(26 GHz) band which is 22.4 dBm [20], when the phased array system has four dual polarized antennas. The first 5G mm-wave antenna module for smartphones uses the antenna array with four dual-polarized antennas [21, 22]. The calculated EIRP using the transmitter front-end is 22.5 dBm (output power + antenna gain(with line loss) + array gain + diversity gain = 3.5 + 4 + 12 + 3 = 22.5). The estimated dual-polarized patch antenna gain with line loss was 4 dB [23]. In addition, since the maximum power reduction of CP-OFDM 16-QAM is 5 dBm, this transmitter sufficiently satisfies the 3GPP minimum peak EIRP specification [20].

### Comparison

The overall power consumption of the proposed 26-GHz transmitter front-end with 1.2 V (IF and mm-wave mixers) and 2.5 V (PPA) DC supplies was 267 mW when an input signal was not applied. The IF mixer, mm-wave mixer, and DA consume 131, 74, and 62 mW, respectively. Table 2 summarizes the measured performance of various mm-wave transmitters. In order to compare the overall performance of transmitters, a figure-of-merit(FoM) including gain, P_*SAT*_, EVM and DC power consumption which are key parameters of the transmitter is justified as follows:

$$\begin{array}{c} \begin{array}{c} FoM=\frac{20log(Gain\left(rms\right)\times P_{SAT}\left(rms\right)/EVM\left(rms\right))}{10log(P_{DC})} \end{array} \end{array}$$ (6)

**Table 2:** Comparison table with CMOS mm-wave transmitters performance.

	[7]	[24]	[25]	[26]	[27]	[28]	[29]	This work
Technology	65 nm CMOS	40 nm CMOS	0.18 *μ*m CMOS	65 nm CMOS	90 nm CMOS	40 nm CMOS	0.13 *μ*m CMOS	65 nm CMOS
Freq. [GHz]	60	60	24	60	60	60	24	26
Gain [dB]	16.4	26	7	15	23	31	30	26.9
OP_1*dB*_ [dBm]	8	-	11	-	3.7	10.8	-	10
P_*SAT*_ [dBm]	13	15.6	14	10.3	8	-	7	14.1
OIP3 [dBm]	-	-	14	-	-	-	-	15.76
EVM [dB]	-20.7	-22	-20.2	-26	-22	-15	-21.9	-17.7
@P_*AVG*_ [dBm]	@6	@12.5	-	-	-	-	-	@3.5
Modulation	16QAM -OFDM	16QAM	BPSK	64QAM	QPSK	16QAM	MSK	16QAM -OFDM
Packaging	flip-chip	no	no	flip-chip	flip-chip	flip-chip	no	flip-chip
Core Area [mm^2^]	9.2 (TX+RX)	0.33	14.3 (w/ PLL)	1.03	1.3	1.5	2.2	2.31
Architecture	heterodyne	direct-conversion	direct-conversion	direct-conversion	direct-conversion	direct-conversion	heterodyne	heterodyne
P_*DC*_ [mW]	207	217	380	168	230	181	118	267
FoM	37.9	55.8	29.4	39.3	37.4	45*	45.2	48.5

*Include OP_1*dB*_ instead of P_*SAT*_

Although there are differences in the operating frequency, the proposed transmitter front-end had quite high P_*SAT*_, OP_1*dB*_, and OIP3, and had the highest P_*SAT*_ and FoM among the flip-chip packaged transmitters.

## Conclusion

A 26-GHz superheterodyne transmitter front-end composed of an IF mixer, a mm-wave mixer, and a pre-power amplifier was designed using 65-nm CMOS technology. The double quadrature architecture was adopted to filter the image frequency without using an image rejection filter. The IF mixer cores were designed with a harmonic rejection technique to eliminate the third-order harmonics of the IF mixer. The load inductors of the IF mixer were designed as a low-Q tunable inductors for a wide-band IF. The pre-power amplifier consisted of a two-stage cascode amplifier and transformers to achieve high output power and sufficient gain. The measured voltage conversion gain, P_1*dB*_, and the saturated output power were 27 dB, 10 dBm, and 14.1 dBm, respectively. The third order distortion suppressed by the HRM was -60.5 dBc at an output power of 10 dBm. The measured EVM for OFDM 16-QAM with a signal bandwidth of 110 MHz was -17.7 dB at an average output power of 3.5 dBm. The total power consumption of the proposed 26-GHz transmitter front-end was 267 mW, and it occupied a chip area of 2.31 mm^2^. The proposed 26-GHz transmitter front-end had a high output power and high conversion gain.
